# Association between baseline LH/FSH and live-birth rate after fresh-embryo transfer in polycystic ovary syndrome women

**DOI:** 10.1038/s41598-021-99850-4

**Published:** 2021-10-14

**Authors:** Nian-jun Su, Cui-yu Huang, Jie Liu, De-ying Kang, Song-lu Wang, Liu-jun Liao, Jin-di Yang, Xi-qian Zhang, Feng-hua Liu

**Affiliations:** 1 Department of Reproductive Health and Infertility, Guangdong Women and Children Hospital, 521 Xingnan Avenue, Panyu District, Guangzhou, 511442 China; 2 Department of Vascular and Endovascular Surgery, Chinese PLA General Hospital, 28 Fuxing Road, Haidian District, Beijing, 100853 China; 3 Department of Evidence Based Medicine and Clinical Epidemiology, West China Hospital, Sichuan University, 37 Guoxuexiang, Chengdu, 610041 China

**Keywords:** Diseases, Reproductive disorders, Infertility

## Abstract

This study aimed to retrospectively analyse the effect of the baseline luteinising hormone/follicle-stimulating hormone ratio (bLH/FSH) on the live-birth rate per fresh-embryo transfer cycle (LBR/ET) in infertile women with polycystic ovary syndrome (PCOS) who received a fresh-embryo transfer. A total of 424 patients with PCOS who underwent the first cycle of in vitro fertilisation (IVF)/intracytoplasmic sperm injection (ICSI) fresh-embryo transfer at our hospital was enrolled. Univariate and multivariate logistic regression analyses, along with curve fitting and a threshold effect analysis, were performed. Baseline LH/FSH levels were a significant (*P* < 0.05) independent risk factor affecting live birth. In the first IVF/ICSI antagonist treatment cycles, LBR/ET after fresh-embryo transfer was relatively flat, until bLH/FSH was 1.0; thereafter, it started to decrease by 17% for every 0.1-unit bLH/FSH increase. Considering the decline in LBR/ET, it is recommended that PCOS women with bLH/FSH > 1.0 carefully consider fresh-embryo transfer during their first IVF/ICSI.

## Introduction

PCOS, the most common endocrinological problem among women of reproductive age, is characterised by chronic ovulatory dysfunction, hyperandrogenism, and infertility. PCOS, affecting 8–13% of women of reproductive age globally^1^, with an incidence of 5.6% in China^2^, has become the main service population for assisted reproductive technology. FSH and LH are both pituitary gonadotropin hormones essential for fertility. Comprising an identical subunit, they are comprised of unique β-subunits, that is, FSHb and LHb, respectively. A decreased frequency of pulsatile gonadotropin-releasing hormone (GnRH) is enabled by FSHb transcription, while an increased frequency is enabled by Lhb transcription. LH stimulates ovarian follicular theca cells to produce androstenedione, whereas FSH stimulates the synthesis of aromatase in granulosa cells, catalysing the conversion of androstenedione to oestradiol^3^. LH and FSH simultaneously act well to stimulate sex steroid secretion and gametogenesis in the ovaries. However, the proper ratio of LH/FSH to coordinate the work between theca cells and granulosa cells to smoothly convert cholesterol to oestradiol without accumulating androgens remains controversial^4^. Studies on the relationship between the LH/FSH cut-off point and pregnancy outcomes have been conducted as early as 1995^5^ because the LH/FSH cut-off point is considered to represent the responsiveness of ovaries to ovulation-stimulating drugs. In the late twentieth century, the LH/FSH cut-off point was 3^5^, but it was decreased to two by the Rotterdam Consensus group in the early twenty-first century^6^. In recent years, the LH/FSH cut-off point is thought to be 1.33^7^. These disputes may also be related to the effect of LHCGR on follicular theca cells and the function of FSHR of granulosa cells^8^. Anti-Müllerian hormone (AMH), also considered to represent PCOS reactivity, is produced in granulosa cells by pre-antral and small antral follicles and is highly correlated to the antral follicle count (AFC) and bLH/FSH in women with or without PCOS^9,10^. It is a negative regulator of early follicular recruitment from the primordial pool^11^. Moreover, the severity of PCOS phenotypes correlates to AMH production, which is higher in anovulatory than in ovulatory patients with PCOS^12^. Although high bLH/FSH and AMH levels in women with PCOS have become a consensus^7^ due to significant disease heterogeneity, neither of them is a diagnostic indicator of PCOS or^7,13^ predictor of the live-birth rate of women with PCOS based on ART^5,14–16^.

Randomised controlled trials have confirmed that, in women with PCOS, the live-birth rate after fresh-embryo transfer is lower than that of frozen-embryo transfer (42.0% vs 49.3%, *P* < 0.05), but frozen-embryo transfer increase the potential risk of eclampsia^17^. Since both fresh and frozen embryos have their own advantages and disadvantages^18^, patients with PCOS with a low risk of OHSS undergoing frozen-embryo transfer may need a longer time to reach pregnancy^19^. In China, patients prefer fresh-embryo over frozen-embryo transfer; therefore, there are no exact indicators to which patients with PCOS are suitable for fresh-embryo transfer. Although E2 and P4 on the trigger day and the number of oocytes retrieved are commonly used for determining cut-off points, they are not precise^5,6,16,20–23^.

Therefore, we assumed a bLH/FSH cut-off point, below which the ovarian reactivity of patients with PCOS is low, and a high live-birth rate after fresh-embryo transfer is expected, which is suitable for fresh-embryo transfer. Above this cut-off point, patients with PCOS have a higher ovarian reactivity, and the live-birth rate after fresh-embryo transfer is consequently expected to be low, which is suitable for frozen-embryo transfer.

## Materials and methods

### Subjects

This retrospective cohort study was performed at the Guangdong Women and Children Hospital, PR, China and was approved by the hospital institutional review board of the Guangdong Women and Children Hospital. The requirement for informed consent was waived due to the retrospective nature of this study. All methods were performed in accordance with the relevant guidelines and regulations. All reports followed the Strengthening the Reporting of Observational Studies in Epidemiology guidelines.

All consecutive infertile women with PCOS aged between 18 and 40 years undergoing their first in vitro fertilisation/intracytoplasmic sperm injection (IVF/ICSI) ovarian stimulation cycle by a GnrH-antagonist (GnRH-ant) protocol between August 2014 and July 2018 were considered eligible patients. Each patient was included only once in the analysis.

Exclusion criteria were a history of ovarian surgery, recurrent miscarriage, endometriosis, congenital or acquired reproductive tract deformities and uncontrolled diseases, such as hypertension, diabetes and thyroid disease. Moreover, we excluded couples who had planned ovarian stimulation for preimplantation genetic testing. Furthermore, patients having cycles with no oocytes retrieved, fertilisation failure, or all frozen-embryo transfer were excluded from analysis.

We obtained data from an independent electronic data system from the Reproductive Center of the Guangdong Women and Children Hospital by searching for PCOS diagnoses and GnRH-ant. A manual medical record review was performed to reduce missing data and validate patient characteristics associated with automated data. We collected the data on all variables as much as possible.

### Treatment plan

All patients underwent ovarian hyperstimulation through a GnRH antagonist protocol. Patients received daily injections of gonadotropins starting on day 2 or 4 of their menstrual cycle or after withdrawal bleeding.

Gonadotropin stimulation was prepared using recombinant follicle-stimulating hormones (Gonal-F, Merck Serono, Italy, or Puregon, Organon, Oss, the Netherlands, or urine highly purified FSH, Menopur, Ferring Pharmaceuticals Ltd., Denmark), with or without human menopausal gonadotropin (hMG; Zhuhai Lizhu Medicine Ltd., China), and recombinant luteinising hormones (rLH, Luveris, Merck Serono Co., Ltd., Switzerland). After using the antagonist, if the patient’s peripheral blood LH was < 1.0 ng/mL, exogenous LH was used.

Gonadotropin initiation was based on patient age, AFC, AMH level, body mass index (BMI) and the experience of the clinician. Initial doses ranged from 75 to 250 IU daily. Four days later, gonadotropin doses were adjusted according to ovarian response, which was assessed by vaginal ultrasound and serum sex hormone levels.

Ganirelix acetate (ganirelix acetate injection; Organon, Netherlands) or cetrotide (cetrorelix acetate injection; Merck Serono, Italy) were introduced at doses of 250–500 μg until the day of trigger, on the fifth day of stimulation, that is, the fixed protocol, or when the dominant follicle diameter reached a mean of 12 mm, that is, the flexible protocol.

When serum LH levels were < 1.0 IU/mL, urine highly purified FSH (Menopur, Ferring Pharmaceuticals Ltd., Denmark), hMG (Zhuhai Lizhu Medicine Ltd.), or rLH (rLH, Luveris, Merck Serono Co., Ltd., Switzerland) at doses of 75 IU daily were to be added if necessary.

Ovulation was triggered with the administration of human or recombinant chorionic gonadotropin (hCG/r-hCG) immediately after the appearance of ≥ two follicles of ≥ 18 mm in diameter or ≥ three follicles of ≥ 17 mm in diameter. Moreover, if at least 60% of follicles were ≥ 15 mm in diameter, urine gonadotropin (hCG, Zhuhai Lizhu Medicine Ltd.) at doses of 8,000/10,000 IU or recombinant gonadotropin (Ovidrel, Merck Serono) at doses of 250 mg was used. If serum E2 was ≥ 5000 pg/mL, GnRH agonist triptorelin (Gonapeptyl, Ferring) at doses of 0.2 mg or GnRH agonist at doses of 0.2 mg combined with urine hCG at doses of 2000 IU was used to reduce OHSS risk^24^. Transvaginal oocyte retrieval was performed 36 h after triggering ovulation. Oocytes collected from each woman were inseminated either via conventional IVF, ICSI, or a combination of both. The corresponding laboratory used a purchased Kato culture medium (KITAZATO, Japan).

Whether to transfer fresh-embryos was decided based on the fertilisation conditions, the E2 level after oocyte retrieval, ascites and endometrial conditions, patient discomfort, and other comprehensive indicators. If the conditions allowed, one or two fresh D3–D6 embryos were subsequently transferred. The morphological evaluation system of oocytes and early embryos is used for embryo cultures by cleaving one or two embryos with at least five blastomeres that are considered to be transferrable embryos; those with 6–10 blastomeres cleaving grade 1 or 2 embryos were considered as high-quality embryos, that is, at least 6 cells with a maximum of 20% fragmentation on day 3. Blastocysts were evaluated according to the Gardner scoring system; embryos graded ≥ 3BB were also considered high-quality.

Luteal phase support was started on the day after oocyte retrieval with 800 mg of intravaginal progesterone capsules daily (Progestan or Organon), 90 mg of gel daily (Crinone, Serono), or 40 mg of intramuscular injection daily (Progesterone) until the day before the pregnancy test. Serum oestradiol and progesterone levels were detected 4 days after transfer and remained at > 200 pg/mL for E2 levels and > 20 ng/mL for P levels; otherwise, E and P were added. If patients were pregnant, the luteal phase support was used until 8 weeks of pregnancy.

### Definition of results and indicators

PCOS diagnosis was re-evaluated according to the Rotterdam criteria^13^; women were considered to have PCOS if they met at least two of the following three criteria, with the exclusion of related disorders: (1) oligo-/anovulation; (2) clinical or biochemical hyperandrogenism, defined as a laboratory-specified elevation in the following androgens: serum T > 75 ng/dL (> 2.6 nmol/L), androstenedione > 3.9 ng/mL (> 13.7 nmol/L) or dehydroepiandrosterone sulphate (DHEAS) > 3.14 ng/mL (> 10.9 µmol/L); and (3) polycystic ovary morphology.

AFC was recorded as the sum of all follicles of 2–9 mm in diameter. Related disorders included congenital adrenal hyperplasia, Cushing syndrome and androgen-secreting tumours.

Baseline sex hormones, including LH/FSH (bLH/FSH), were initiated at the first visit of the patient and one year before the start of the cycle. These hormones were detected in the first 2–5 days of the cycle of patients with spontaneous menstrual cramps or progesterone withdrawal bleeding. If oral contraceptives (OCs) had been used for treating PCOS in another hospital, we would ask to stop administration for at least one month as a washout period. If there were multiple inspection reports, the report closest to the start-up time prevailed.

The live-birth rate per fresh-embryo transfer cycle (LBR/ET) was analysed as the primary outcome using the following calculation: live-birth rate = (number of live-birth cycles/number of all transfer cycles) × 100. Other outcomes included the clinical pregnancy rate, spontaneous abortion rate, early miscarriage rate, ongoing pregnancy rate and moderate-to-severe OHSS/fresh-embryo transfer (msOHSS/ET).

The overall fertilisation rate was calculated by dividing two pronuclear stage embryos by the total number of oocytes obtained. For patients in whom ICSI was used, the fertilisation rate was calculated by dividing two pronuclear stage embryos by the total number of mature oocytes.

We used the American Society for Reproductive Medicine 2017 consensus definitions to define clinical outcomes^25^. The implantation rate was calculated as the number of gestational sacs visualised on transvaginal ultrasound divided by the number of embryos transferred. Serum hCG levels were measured 14 days after ET, and a value above 5 IU/mL was considered to indicate test positivity. CRP levels were calculated as the presence of foetal cardiac activity, confirmed by transvaginal ultrasound per ET. The spontaneous abortion rate was defined as pregnancy loss after the sonographic visualisation of an intrauterine gestational sac per ET. Live-birth rates were considered to increase if at least one living child was delivered.

The OHSS classification was based on the Golan criteria^26^. Patients were considered to have high-risk OHSS if they met one of the following criteria: (1) E2 level ≥ 5000 pg/mL on the day of hCG administration, (2) E2 level ≥ 1500 pg/mL on the day after oocyte retrieval, (3) number of oocytes retrieved ≥ 25, and (4) E2 level between 3000 and 5000 pg/mL on the day of hCG administration or between 1000 and 1500 pg/mL on the day after oocyte retrieval, as well as large-diameter ovaries and obvious effusion in the pelvic cavity on the day of transfer. In addition to freezing all embryos, some patients with a high risk of OHSS were also given albumin as a plasma expander following oocyte retrieval to prevent early-onset OHSS.

### Statistical analysis

The baseline and cycle characteristics and treatment outcomes of all patients were analysed. To evaluate the impact of bLH/FSH, patients were categorised into three groups according to their bLH/FSH: < 1, 1–2.49, and ≥ 2.5. Continuous data are presented as mean ± standard deviation or median (Q1–Q3) values depending on distribution normality. Normality was examined using the Shapiro–Wilk test. In addition, categorical data were presented as the number of cases and the corresponding percentage. Categorical data and continuous data that did not show a normal distribution were analysed using Pearson’s chi-squared test, Fisher’s exact test, or Kruskal–Wallis test, as appropriate. A *P*-value < 0.05 was considered statistically significant. We adjusted for potential confounding factors if one of the following criteria were present: (1) literature-based confounders, (2) *P* < 0.1 in univariate analyses, and (3) effect value changes in covariate screenings > 10%. Based on the results found in previous literature, age and BMI were selected for the multivariate regression analysis^7,20,27–29^. Univariate regression analyses were performed for all variables to identify candidate factors for predicting primary LBR/ET outcomes. Variables, including ‘endometrial thickness on transfer day’ and ‘progesterone on initiation day’, showing a tendency to be associated with LBR/ET (*P* < 0.10) in the univariate analysis, were selected for the multivariate model. A multivariate stepwise logistic regression analysis was performed with LBR/ET as the dependent variable and bLH/FSH as the main independent variable. The forward selection method was used to include variables with a *P*-value < 0.05 in the multivariate model. Both the non-adjusted and multivariate-adjusted models were used. Subsequently, smooth-curve fitting and threshold saturation effect analyses were performed; the likelihood of LBR/ET was described as an odds ratio (OR) with a standard error and 95% confidence intervals (95% CIs). Statistical significance was set at a two-tailed α value of 0.05. Splines were fit using a logistic regression model using the generalised additive model; corresponding adjustments were made in the multivariate-adjusted model. All analyses were performed using the R Statistical Software (http://www.R-project.org, The R Foundation) and Free Statistics analysis platform.

### Ethics

The personal information of patients was not used in this retrospective cohort study. This study was approved by the Medical Ethics Committee of Guangdong Women and Children Hospital (No. 202101041), and the hospital’s institutional review board approved the study and waived the requirement for obtaining informed consent due to the retrospective nature of this study. All reports followed the Strengthening the Reporting of Observational Studies in Epidemiology guidelines.

## Results

### General conditions and pregnancy outcomes

**Figure 1:**
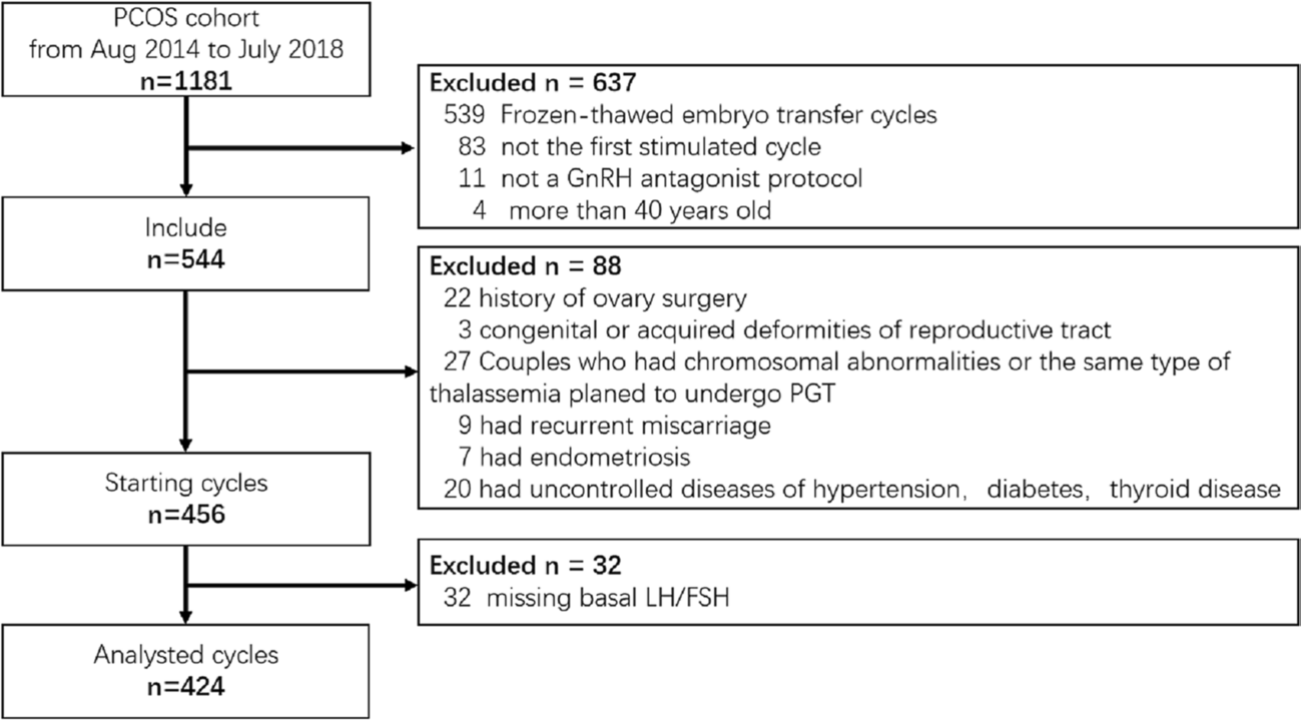
Flow chart of relationship baseline luteinizing hormone/follicle-stimulating hormone ratio and live birth rate per fresh-embryo transfer cycle in women with polycystic ovary syndrome in their first in vitro fertilization/intracytoplasmic sperm injection cycle.

Of the 1,181 consecutive patient cycles between August 2014 and July 2018, 802 were excluded from this study. Thus, 424 starting cycles, including 169 fresh-embryo transfer cycles, were included in the analysis (Fig. 1). Double embryo transfer comprised 94.7% of cases. There were 110 cases of clinical pregnancy, including 6 cases of ectopic pregnancy and 104 cases of intrauterine pregnancy. There were 8 cases of early miscarriage among intrauterine pregnancies. Finally, there were 96 cases of continued pregnancy, including 6 cases of late abortion and 90 cases of live birth. In live birth cases, 26 cases included preterm labour, and 64 cases included on-term labour. Total LBR/ET was 53.3% (90/169), and LBR/S was 21.2% (90/424). The clinical multiple gestation rate was 40.9% (45/110), and the multiple fetuses live birth rate was 35.6% (32/90) (Supplementary Table 1). Conversely, 255 patients did not undergo fresh-embryo transfer because of a high risk of OHSS and the freezing of all embryos (195 patients); or low response, lack of good embryos to transfer, or other reasons (60 patients). Overall, 14 of 169 fresh ET patients (8.3%) presented with msOHSS/ET (Table 1).

**Table 1:** Demography and clinical pregnancy outcomes of the patients.

Group	Total	< 1	1–2.49	≥ 2.5	*P*
N	424	131	246	47	
Age (years)	29.2 ± 3.6	29.5 ± 4.0	29.1 ± 3.3	28.9 ± 4.2	0.521^a^
Infertility duration (years)	3.0 (2.0–5.0)	3.0 (2.0–5.0)	3.0 (2.0–5.0)	3.0 (2.0–5.0)	0.799^b^
BMI (kg/m^2^)	22.8 ± 3.4	23.3 ± 3.5	22.8 ± 3.3	21.4 ± 2.7	0.004^a^
bAMH (ng/mL)	11.5 ± 5.4	9.4 ± 4.5	12.4 ± 5.4	13.5 ± 6.2	< 0.001^a^
AFC	23.3 ± 5.1	22.3 ± 5.5	23.5 ± 4.7	24.9 ± 5.0	0.006^a^
bLH/FSH	1.5 ± 0.8	0.8 ± 0.2	1.6 ± 0.4	3.1 ± 0.8	< 0.001^a^
bFSH	6.2 ± 1.5	6.3 ± 1.5	6.2 ± 1.5	5.7 ± 1.7	0.060^a^
bLH	9.1 ± 5.0	4.7 ± 1.5	9.8 ± 3.7	17.4 ± 5.1	< 0.001^a^
bT (ng/mL)	0.4 ± 0.2	0.3 ± 0.2	0.4 ± 0.2	0.5 ± 0.2	< 0.001
Androstenedione(nmol/L)	11.9 ± 5.4	9.7 ± 3.9	12.5 ± 5.4	14.0 ± 6.8	0.001
Free androgen index	3.6 ± 3.2	2.7 ± 1.9	4.1 ± 3.9	3.6 ± 1.9	0.025
sLH/FSH	1.1 (0.9, 1.8)	0.9 (0.6, 1.2)	1.3 (1.0, 1.8)	1.5 (1.1, 2.4)	< 0.001^b^
sP4 (ng/mL)	0.42 (0.23, 0.60)	0.43 (0.23, 0.63)	0.42 (0.22, 0.60)	0.36 (0.25, 0.54)	0.646
Total dose of gonadotrophins (ampoule^d^)	19.3 ± 7.3	21.0 ± 7.9	18.9 ± 7.2	16.4 ± 4.9	< 0.001^a^
Duration of ovarian stimulation (days)	10.6 ± 2.2	10.7 ± 2.1	10.7 ± 2.3	9.9 ± 1.6	0.061^a^
tE2 (pg/mL)	4633.6 ± 2950.1	3799.8 ± 2254.9	4850.5 ± 3059.6	5822.6 ± 3482.5	< 0.001^a^
NOR	19.0 ± 11.2	16.1 ± 9.6	20.1 ± 11.9	21.7 ± 10.1	0.001^a^
Endometrial thickness on transfer day (mm)	10.0 ± 2.3	10.1 ± 2.3	10.1 ± 2.2	9.0 ± 2.0	0.196^a^
**Embryo age on transfer day (d)**					0.184^c^
3	157/169 (92.9)	57/63 (90.5)	88/92 (95.7)	12/14 (85.7)	
5	12/169 ( 7.1)	6/63 (9.5)	4/92 (4.3)	2/14 (14.3)	
**Number of embryo transfer**					0.062^c^
1	9/169 ( 5.3)	5/63 (7.9)	2/92 (2.2)	2/14 (14.3)	
2	160/169 (94.7)	58/63 (92.1)	90/92 (97.8)	12/14 (85.7)	
CPR	110/169 (65.1)	44/63 (69.8)	58/92 (63.0)	8/14 (57.1)	0.539^c^
Early miscarriage rate	8/110 ( 7.3)	3/44 (6.8)	5/58 (8.6)	0/8 (0)	1.000^c^
OPR	96/169 (56.8)	41/63 (65.1)	48/92 (52.2)	7/14 (50.0)	0.243^c^
Moderate–severe OHSS rate	14/169 ( 8.3)	4/63 (6.3)	9/92 (9.8)	1/14 (7.1)	0.821^c^
LBR/ET	90/169 (53.3)	39/63 (61.9)	44/92 (47.8)	7/14 (50.0)	0.218^c^

*BMI* Body Mass Index, *bAMH* Anti-Müllerian facto, *AFC* antral follicle count, *bLH/FSH* baseline luteinizing hormone/follicle stimulating hormone ratio, *bFSH* baseline follicle stimulating hormone, *bLH* baseline luteinizing hormone, *bT* baseline testosterone, *sLH/FSH* luteinizing hormone/follicle stimulating hormone ratio on started day, *sP4* progesterone on started day, *tE2* estradiol on triger day, *NOR* Number of oocytes retrieved, *CPR* clinical pregnancy rate, *OPR* ongoing pregnancy rate, *LBR/ET* Live-birth rate per fresh-embryo transfer cycle.

^a^Values are mean + SD.

^b^Values are median (IQR).

^c^Fisher test. Values are number/total number (percentage).

^d^75 IU per ampoule.

The baseline characteristics, controlled ovarian stimulation progress and clinical outcomes of all participants, which were divided into three groups according to bLH/FSH, are presented in Table 1. Among the 424 infertile women with PCOS, 30.9% (131/424) had a bLH/FSH < 1 (group A), 58.0% (246/424) had a bLH/FSH of 1–2.49 (group B), and 11.1% (47/424) had a bLH/FSH ≥ 2.5 (group C). Baseline AMH levels, AFC, bLH/FSH, bLH, baseline oestradiol levels, baseline testosterone levels, androstenedione, free androgen index, total dose of gonadotropins (ampoule), duration of ovarian stimulation (days), and the number of oocytes retrieved gradually increased in the three groups (*P* < 0.05). BMI gradually declined in all groups (*P* = 0.004). However, age, infertility duration, bFSH, progesterone on the start day (sP4), embryo age on transfer day, number of transfer embryos, live-birth rate per fresh-embryo transfer cycle, and msOHSS rate were stable in all groups *(P* > 0.05).

### Logistic regression analysis

A univariate logistic regression analysis demonstrated the potential confounders on the live-birth rate (*P* < 0.10), including bLH/FSH, bFSH, sP4, and endometrial thickness on transfer day (Table 2). After collinearity screening, bFSH levels were excluded. Thereafter, age, BMI, endometrial thickness on transfer day and sP4 were selected (Table 2) and adjusted in the multivariable logistic regression analysis (Table 3). The unadjusted odds ratio (OR) between bLH/FSH and LBR/ET was 0.954 (0.912 and 0.998, respectively; *P* = 0.042). After adjusting for age and BMI, the adjusted OR (aOR) was 0.949 (0.906 and 0.995, respectively; *P* = 0.029) (Table 3). After adjusting for age, BMI and endometrial thickness on transfer day, aOR was 0.954 (0.910 and 1.001; *P* = 0.053), and after adjusting for age, BMI, endometrial thickness on transfer day and sP4, aOR was 0.952 (0.899 and 1.008; *P* = 0.091). ORs were robust between unadjusted and adjusted models.

**Table 2:** Univariate and multivariate analysis the live-birth rate per embryo transferred cycle according to the baseline luteinizing hormone/follicle stimulating hormone ratio.

	Univariate analysis		Multivariate analysis	
	OR (95%CI)	*P*	OR (95%CI)	*P*
Age (years)	0.99 (0.91, 1.07)	0.794	1.01 (0.91, 1.11)	0.864
BMI (kg/m2)	0.97 (0.89, 1.06)	0.541	0.96 (0.85, 1.08)	0.482
bAMH (ng/mL)	1.00 (0.93, 1.07)	0.940		
AFC	1.04 (0.97, 1.11)	0.234		
bLH/FSH^a^	0.95 (0.91, 1.00)	0.042	0.96 (0.90, 1.01)	0.144
bFSH	1.20 (0.98, 1.46)	0.074	1.23 (0.95, 1.60)	0.121
bLH	0.96 (0.9, 1.02)	0.197		
bT (ng/mL)	0.19 (0.03, 1.38)	0.102		
Androstenedione (nmol/L)	0.96 (0.87, 1.06)	0.468		
DHEAS (ug/dL)	1.00 (1.00, 1.00)	0.443		
SHBG (nmol/L)	1.00 (0.99, 1.00)	0.423		
FAI	0.97 (0.81, 1.16)	0.734		
HOMA index	1.2 (0.86, 1.67)	0.278		
TC (mmol/L)	0.90 (0.70, 1.16)	0.423		
UA (umol/L)	1.00 (0.99, 1.00)	0.478		
sE2 (pg/mL)	0.99 (0.97, 1)	0.151		
sP4 (ng/mL)^a^	0.81 (0.70, 0.94)	0.006	0.79 (0.67, 0.92)	0.003
Started gonadotropin dose (ampoule)	1.00 (0.99, 1.00)	0.583		
Total dose of gonadotrophins (ampoule)	1.00 (0.99, 1.01)	0.691		
tE2 (pg/mL)	1.00 (1.00, 1.00)	0.192		
NOR	0.97 (0.91, 1.03)	0.305		
Endometrial thickness on transfer day (mm)	1.19 (1.03, 1.37)	0.020	1.25 (1.04, 1.49)	0.016
Embryo age on transfer day (d)	1.12 (0.62, 2.03)	0.715		
Number of embyro transfer	0.91 (0.23, 3.5)	0.887		

*BMI* Body Mass Index, *bAMH* Anti-Müllerian facto, *AFC* antral follicle count, *bLH/FSH* baseline luteinizing hormone/follicle stimulating hormone, *bFSH* baseline follicle stimulating hormone, *bLH* baseline luteinizing hormone, *bT* baseline testosterone, *DHEAS* Dehydroepiandrosterone sulfate, *SHBG* Sex hormone binding globulin, *FAI* Free androgen index, *TC* triglycerides, *UA* uric acid, *sE2* estradiol on started day, *sP4* progesterone on started day, *tE2* estradiol on triger day, *NOR* Number of oocytes retrieved. *OR* odds ratio, *CI* confidence interval.

^a^per change 0.1 unit.

**Table 3:** Multivariate analysis on the live-birth rate per fresh-embryo transfer cycle according to the baseline luteinizing hormone/follicle stimulating hormone ratio.

	Non-adjusted, OR (95% CI)	Adjust I, OR (95% CI)	Adjust II, OR (95% CI)	Adjust III, OR (95% CI)
bLH/FSH^a^	0.954 (0.912, 0.998)	0.949 (0.906, 0.995)	0.954 (0.910, 1.001)	0.952 (0.899, 1.008)
*P*	0.042	0.029	0.053	0.091

*OR* odds ratio, *CI* confidence interval, *Adjust I* adjust age and body mass index, *Adjust II* Adjust I + endometrial thickness on transfer day, *Adjust III* Adjust II + progesterone on started day. ^a^per change 0.1 unit.

### Threshold effect analysis

**Figure 2:**
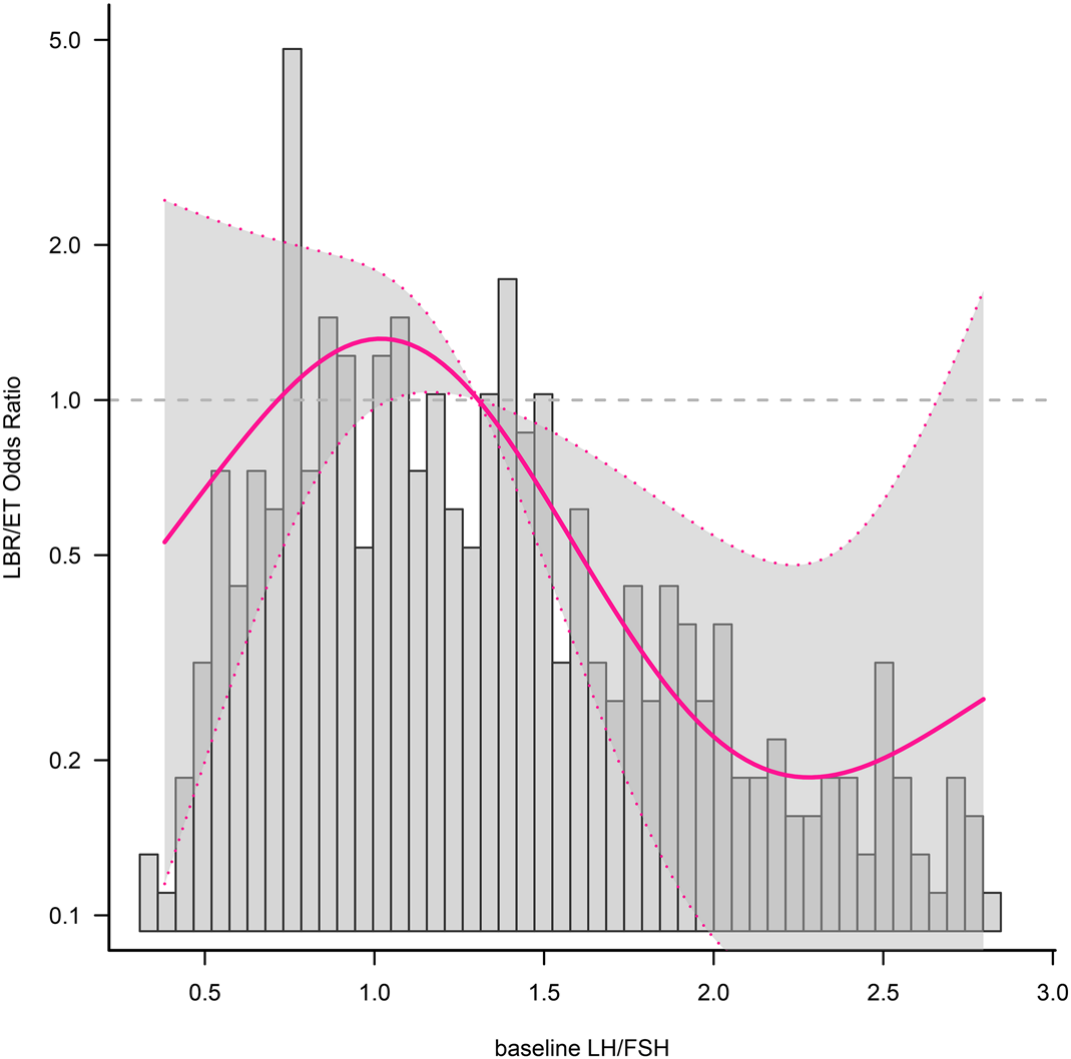
Smooth curve fitting of relationship between baseline luteinizing hormone/follicle-stimulating hormone ratio and live-birth rate per fresh-embryo transfer cycle in women with polycystic ovary syndrome in their first in vitro fertilization/ intracytoplasmic sperm injection cycle. The solid line and dashed line represent the estimated values and their corresponding 95% confidence intervals. Only 95% of the data is displayed. Adjusted for age and BMI.

The association between bLH/FSH and LBR/ET was not linear but curved. After adjusting for confounding factors, including age, BMI and endometrial thickness on transfer day, LBR/ET was relatively flat, until bLH/FSH was 1.0; it thereafter started to decrease by 17% (aOR: 0.83; and 95% CI: 0.75 and 0.92) for every 0.1-unit bLH/FSH increase (*P* for nonlinearity < 0.001, Table 4). When bLH/FSH was beyond 2.5, LBR/ET did not significantly change (Fig. 2).

**Table 4:** Threshold effect analysis of relationship of baseline luteinizing hormone/follicle stimulating hormon ratio (per change 0.1 unit) on live birth rate per fresh-embryo transfer cycle.

	Adjusted OR (95% CI)	*P*
One-line linear regression model	0.95 (0.91, 1.00)	0.029
**Three-piecewise linear regression model**		
bLH/FSH < 1.0	1.20 (0.93, 1.55)	0.171
bLH/FSH ≥ 1.0, < 2.5	0.83 (0.75, 0.92)	< 0.001
bLH/FSH ≥ 2.5	1.18 (0.85, 1.65)	0.319
Log-likelihood ratio test		< 0.001

*OR* odds ratio, *CI* confidence interval, *bLH/FSH* baseline luteinizing hormone/follicle stimulating hormone ratio. bLH/FSH per change 0.1 unit. Adjust for age, body mass index.

## Discussion

We found that in infertile women with PCOS aged between 18 and 40 years, when IVF/ICSI combined with the antagonist program was first performed and bLH/FSH was < 1.0, the pregnancy rate of fresh-embryo transfer was more stable and higher. LBR/ET was relatively flat until bLH/FSH was 1.0; it subsequently started to decrease by 17% for every 0.1-unit increase in LBR/ET (*P* for nonlinearity < 0.001). bLH/FSH has a nonlinear relationship with the live-birth rate of fresh-embryo transfer.

The two-cell theory suggests that both FSH and LH are required for normal follicular growth. These hormones are different but have related commands that act on two important cells before and after follicle formation, which are follicular membrane and granulosa cells. Therefore, their ratio at different time points during the menstrual cycle is extremely important. In each natural cycle, the ratio between LH and FSH also changes to produce different amounts of androgens, oestrogen and progesterone. Baseline LH/FSH on days 3–5 of the menstrual cycle indicates that after the dominant follicle is recruited, the dominant follicle continues to mature under FSH. However, in infertile women with PCOS, an increase in the frequency and amplitude of the secretion of GnRH leads to an increase in the frequency and amplitude of the secretion of LH secretion^30^. Increased LH levels trigger ovarian follicular theca cells to secrete excess levels of androgen, whereas FSH stimulates granulosa cells in the ovary to convert extra androgens to oestrogen^31^. High bLH/FSH levels may impair the formation of ovarian follicles^5^, resulting in abnormal granular cell function^32^. Patients in our study with a high bLH/FSH also had a higher AFC and increased testosterone levels, which is consistent with the findings of Yu Ng et al.^33^, who also described a possible correlation between AFC, LH and testosterone. Cimino et al. even proposed the theory that PCOS originates from increased ovarian AMH^34^. Our study confirmed the abovementioned hormonal pathological changes. When bLH/FSH increased, bLH, baseline oestradiol, baseline testosterone, baseline androstenedione, AMH and AFC contents all increased, and LBR/ET decreased. Although the current diagnostic standard regarding PCOS does not include serum bLH or bLH/FSH levels^13,15^, past studies have shown that high bLH levels may have a negative impact on IVF/ICI treatment results^35^. In this study, we confirmed the negative impact of bLH/FSH on LBR/ET.

In the 1990s, the baseline LH/FSH cut-off point for PCOS was recognised to be 3^5^. Subsequently, many studies have set the normal cut-off point of LH/FSH to be 2^6,20,21,23^. For example, Esmaeilzadeh et al. believed that setting the cut-off point of LH/FSH as 2, that of FSH as 7 and that of LH as 10 best reflects the clinical symptoms of PCOS^20^. However, other scholars believe that an LH/FSH cut-off value of 1.5 is more appropriate^22^. Even with IVM, the clinical pregnancy rate of the group with a cut-off value of > 1.5 is still lower than that of the group with a cut-off value of 0.5–1.5^22^. Interestingly, we observed that when the baseline LH/FSH ratio was < 1.0, the live-birth rate after fresh-embryo transfer was stable at a high level. However, when bLH/FSH was between 1 and 2.5, for every 0.1 unit this ratio increases, the aforementioned rate decreases by 17%. Therefore, we believe that the cut-off point of bLH/FSH should be 1.0 to be associated with LBR/ET.

Accordingly, it is essential to perform pre-treatment procedures before assisted reproductive technology treatment. Baseline LH/FSH and LH decreased after oral contraceptive (OC) treatment^6,36^ and progestin administration^5^. The administration of multivitamins for 3 months significantly reduced LH/FSH from 2.5 to 1.9^37^. Therefore, it is worthwhile to conduct further comparative studies between methods to provide the best clinical plan for reducing bLH/FSH.

We found that bLH/FSH in patients with PCOS was > 1.0 and that the live-birth rate after fresh-embryo transfer consequently decreased. Simultaneously, if the LH/FSH of patients is not pre-treated or not corrected after pre-treatment, fresh-embryo transfer may be cancelled for the sake of patients because studies have shown that the live-birth rate after frozen-embryo transfer is significantly higher than that of fresh-embryo transfer in patients with PCOS (49% vs 42%)^17^. However, whether the live-birth rate after frozen-embryo transfer is higher than that of fresh-embryo transfer in patients with PCOS with a bLH/FSH > 1.0 remains to be further studied.

In this retrospective cohort study, 95% of patients underwent double-embryo transfer, resulting in a twin delivery rate of 35.6%. It is well known that elective single-embryo transfer (e-SET) can significantly reduce the live birth rate and multiple live birth rate^38^. Because ART patients are treated at their own expense in mainland China, as well as the one-child and two-child policy (starting on October 29, 2015) being fully implemented for a long time, the patients strongly request double-embryo transfer to maximise their live birth rate^17,39^. With the liberalisation of the three-child policy and the ART patients gradually accepting the concept that e-SET is safer for children, we will implement the e-SET policy. In the future, when the sample size is sufficient, we will verify this conclusion for eSET in patients with PCOS.

The advantages of this research were the appropriate statistical methods to determine the curve relationship of the association between bLH/FSH and LBR/ET, instead of a simple straight-line relationship^5^. Moreover, we found the inflexion point of the smooth curve fitting instead of manual grouping^40^. However, this study has some limitations. First, some patients with bLH/FSH were tested in other hospitals, while others were tested in our hospital, and this may have induced a bias during testing. However, we carefully checked whether the third to fifth days of the menstrual cycle were tested; the unit of measurement in the test report was converted, if necessary. Second, we did not collect few confounding factors, such as the Ferriman-Gallway hair score, PCOS phenotypes and thyroid-stimulating hormone (TSH) levels. However, these confounding factors have no practical application value in assisted reproductive technology clinics. Meanwhile, TSH levels were reviewed preoperatively, and hyperstimulation was allowed only when it was normal. Third, there were no records of OC medication before the bLH/FSH was detected. At the first visit, if OCs had been used for treating PCOS in an outside hospital, we would ask to stop administration for at least one month as a washout period. Fourth, a limitation of our study was its retrospective nature. There were 7% missing bLH/FSH data. However, a sensitivity analysis confirmed that these data were missing at random and did not affect the main results. Fifth, we only included patients undergoing fresh-embryo transfer, and the results could not be extrapolated to frozen-embryo transfer cycles. In patients with LH/FSH > 1.0, whether the live-birth rate after frozen-embryo transfer is higher than that of fresh-embryo transfer remains to be further elucidated.

In conclusion, considering the decline in LBR/ET, it is recommended that PCOS women with bLH/FSH > 1.0 carefully consider fresh-embryo transfer during their first IVF/ICSI. A large-scale study is needed to confirm our findings, explain whether pre-treatment is required, and determine the best pre-treatment method.

## Supplementary Information

Supplementary Information.
