# Supplementary material for: Association between baseline LH/FSH and live-birth rate after fresh-embryo transfer in polycystic ovary syndrome women
Source: Sci Rep. 2021 Oct 14;11:20490. doi: 10.1038/s41598-021-99850-4 (PMC8516967; doi:10.1038/s41598-021-99850-4)
Supplement: Supplementary file 1 — Supplementary Information. [file 41598_2021_99850_MOESM1_ESM.docx]

|  | | | | | | |
| --- | --- | --- | --- | --- | --- | --- |
|  | single-embryo transfer | | double-embryo transfer | | | Total |
| fresh-embryo transfer cycle number | 9 | | 160 | | |  |
| implanted embryo number | 0 | 1 | 0 | 1 | 2 |  |
| implanted number (rate) | 4(44.4) | 5(55.6) | 55/160(34.4) | 60/160(37.5) | 45/160(28.1) |  |
| clinical pregnancy rate |  |  |  |  |  | (5+60+45)/169(65.1) |
| clinical multiple gestation rate |  |  |  |  |  | 45/(5+60+45)(40.9) |
| early abortion |  |  |  | 13 | 1 |  |
| intra-uterine |  | 0 |  | 7 | 1 |  |
| extra-uterine |  | 0 |  | 6 | 0 |  |
| ongoing pregnancy |  | 5 |  | 47 | 44 |  |
| ongoing multiple gestation rate |  |  |  |  |  | 44/(5+47+44)(45.8) |
| late abortion |  | 0 |  | 3 | 3 |  |
| live birth |  | 5 |  | 44 | 41 |  |
| single fetuses |  | 5 |  | 44 | 9 |  |
| Premature delivery |  | 1/5(20.0) |  | 7/44(15.9) | 2/9(22.2) |  |
| Full-term birth |  | 4/5(80.0) |  | 37/44(84.1) | 7/9(77.8) |  |
| multiple fetuses |  |  |  |  | 32 |  |
| Premature delivery |  |  |  |  | 16/32(50.0) |  |
| Full-term birth |  |  |  |  | 16/32(50.0) |  |
| multiple fetuses live birth rate |  |  |  |  |  | 32/(5+44+41)(35.6) |

**Supplementary Table 1** pregnancy outcomes of multiple gestation and multiple fetuses
